# Experimental Evaluation of ^65^Zn Decorporation Kinetics Following Rapid and Delayed Zn-DTPA Interventions in Rats. Biphasic Compartmental and Square-Root Law Mathematical Modeling

**DOI:** 10.3390/pharmaceutics13111830

**Published:** 2021-11-02

**Authors:** Victor Voicu, Marilena Jiquidi, Constantin Mircioiu, Roxana Sandulovici, Adrian Nicolescu

**Affiliations:** 1 Department of Clinical Pharmacology and Toxicology, Faculty of Medicine, Carol Davila University of Medicine and Pharmacy, 050477 Bucharest, Romania; victorvoicu@acad.ro; 2 Army Center for Medical Research, 021051 Bucharest, Romania; victor.voicu@yahoo.com; 3 Laboratory of Radiobiology, Fundeni Clinical Institute, 022328 Bucharest, Romania; 4 Faculty of Pharmacy, Carol Davila University of Medicine and Pharmacy, 020956 Bucharest, Romania; 5 Faculty of Pharmacy, Titu Maiorescu University, 040441 Bucharest, Romania; 6 Department of Medicine, Queen’s University, Kingston, ON K7L 3N6, Canada; adrian.nicolescu@yahoo.com

**Keywords:** radionuclides, kinetics of decorporation, compartmental vs. diffusional models, square-root law

## Abstract

The decorporation kinetics of internal radionuclide contamination is a long-term treatment raising modeling, planning, and managing problems, especially in the case of late intervention when the radiotoxic penetrated the deep compartments. The decorporation effectiveness of the highly radiotoxic ^65^ZnCl_2_ by Zn-DTPA (dosed at 3.32 mg and 5 mg/0.25 mL/100 g body weight) was investigated in Wistar male rats over a ten-day period under various treatments (i.e., as a single dose before contamination; as a single dose before and 24 h after contamination; and as daily administrations for five consecutive days starting on day 12 after contamination). The radioactivity was measured using the whole-body counting method. Mono- and bi-compartmental decorporation kinetics models proved applicable in the case of a rapid intervention. It was found that a diffusion model of the radionuclide from tissues to blood better describes the decorporation kinetics after more than ten days post treatment, and the process has been mathematically modeled as a diffusion from an infinite reservoir to a semi-finite medium. The mathematical solution led to a square-root law for describing the ^65^Zn decorporation. This law predicts a slower release than exponential or multiexponential equations, and could better explain the very long persistence of radionuclides in the living body. Splitting data and modeling in two steps allows a better understanding, description and prediction of the evolution of contamination, a separate approach to the treatment schemes of acute and chronic contamination.

## 1. Introduction

Accidents at nuclear power plants are thought highly improbable by adepts of nuclear energy, although a zero risk is unattainable. The 1979 Three Mile Island accident and the Chernobyl accident in 1986 were real accidents with consequences evidenced long after and farther from the accident sites. Natural catastrophes can also completely change the problem. For example, Japan was hit on 11 March 2011 by the great East Japan earthquake followed by a catastrophic tsunami which caused the Fukushima Daiichi nuclear power plant disaster. Additionally, terrorist actions throughout the world underscore the growing threat of radiological terrorism. Exposure to radionuclides could result in internal contamination of a significant number of individuals [1]. The most critical health issue of the radiotoxicity when considering the effects on children is an increased incidence of the thyroid cancer, as demonstrated in people who were children or adolescents at the time of the Chernobyl accident [2].

The available effective agents decorporate a limited range of radionuclides. Nevertheless, their pharmaceutical formulation makes their administration challenging in mass casualty situations [3]. The efficacies of the medical decorporation strategies highly depend on the time of the treatment delivery after intake. Thus, the first hour and no later than 3–4 h post exposure are optimal when attempting to remove the radionuclides from the extracellular fluids prior to cellular uptake [4].

Ethylenediaminetetraacetic acid (EDTA) and diethylenetriaminepentaacetic acid (DTPA) were introduced in the 1950’s. DTPA is currently clinically approved by FDA for treating internal contamination with transuranium elements [5], but its activity after oral administration is low and it is formulated only as intravenous injections as Ca^2+^ (Ca-DTPA) or Zn^2+^ (Zn-DTPA) chelates to avoid toxicity. DTPA chelates act on the serum calcium metabolism that induces functional damages to the cardiovascular system [6]. Alternatively, intravenous injection and concomitant inhalation of DTPA powder have been tried in lung contamination [7,8,9,10].

The usual mathematical models belong to two extremes: the mono-compartmental elimination model, with limited performances, and the multiple compartmental model, with unstable mathematical solutions. The development of a biokinetic model describing the mechanisms of actinide decorporation with DTPA was initiated as a task in the European Coordinated Network on Radiation Dosimetry (CONRAD) [11]. The major limitation of this model arises from the large number of parameters which causes a lack of stability (i.e., small fluctuations of the entering data generate large variability of the estimated parameters) [12].

The present research investigated the effects of Zn-DTPA on the decorporation kinetics of the highly radiotoxic ^65^ZnCl_2_ over a ten-day interval. ^65^Zn is an activation product obtained in testing “conventional” atomic bombs [13]. Ca-DTPA and Zn-DTPA have been used investigatory for over 40 years to accelerate the excretion of plutonium (Pu) and americium (Am) from the body. In our research we selected ^65^Zn instead of Pu or Am, because it is a gamma emitter, and the activity can be easily measured by counting the whole body. Another advantage is that it has a much shorter half-life than Pu and Am.

Zinc is one of the most abundant trace elements in humans. It is normally found in all tissues and is a cofactor in many enzyme systems. Muscles and bones contain about 90% of the total amount of zinc in the body [14]. After feeding rats with zinc acetate for 3 months, elevated zinc levels were significant in the heart, spleen, kidneys, liver, bones, and blood [15]. The highest increases were in bone (258% of control value) and blood (520% of control value) [15]. Chelators bind trace elements and especially endogenous Zn [16]. Thus, Zn-DTPA was considered as an alternative to Ca-DTPA for longer treatments.

The data analysis aimed at finding simple, stable, and easy applicable mathematical models to describe the whole-body decontamination following immediate and delayed treatments with Zn-DTPA. The proposed diffusional model fitted well our experimental results, particularly for delayed therapeutic interventions after contamination, which can represent a potential advantage over the most common cases that currently are seen in practice.

## 2. Materials and Methods

### 2.1. Chemicals

^65^ZnCl_2_ solution was purchased from Amersham and had an activity of 4.42 MBq/mL. Zn-DTPA was synthesized at the Romanian National Institute for Chemistry and Drugs.

### 2.2. Equipment

The radioactivity of the contaminated animals was measured by the whole-body counting method using a spectrometric Tracor Northern TN-1705 analyzer with 1024 channels, a 4 inch × 4 inch NaI (TI) crystal for a period of 100 s/ rat.

### 2.3. Animals Studies

The whole-body radioactivity measurements were performed in three different settings on male Wistar rats supplied by the Fundeni Clinical Hospital vivarium. The animal use procedures were in accordance with the recommendations of the European Union Council 86/609 EEC [17]. After the reception in the laboratory, the rats were kept for seven days in the accommodation with the new habitat, with standard conditions of air, light, water, and temperature. The animal contaminations with ^65^ZnCl_2_ and treatments with Zn-DTPA were performed by intraperitoneal injections (i.p.)

**Figure 1:**
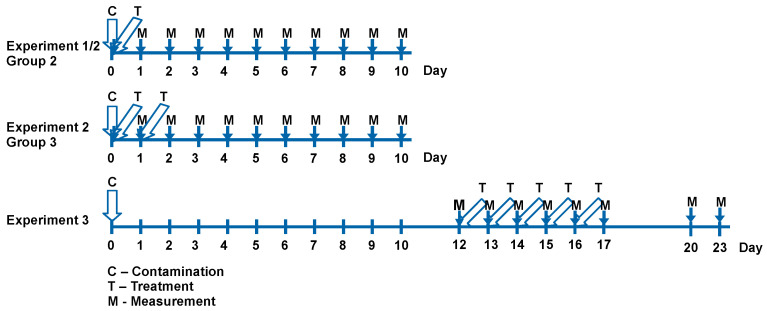
Schematic representation of the experimental methods.

Zn-DTPA was used at a dose of 3.3 or 5 mg/100 g body weight, i.e., 33 or 50 mg/kg. The usual recommended dose in patients is 1000 mg once a day in adults, which corresponds to about 15 mg/kg for 70 kg body weight. An amount of 25–50 mg/kg is recommended for children. Thus, the doses used in the experiments correspond approximately to the recommended clinical doses. It were performed three different experiments (Figure 1).

Experiment 1 involved two groups of seven rats. Group 1 (control 1) received i.p. 0.037. MBq ^65^ZnCl_2_/rat. Group 2 received i.p. solutions containing 3.32 mg (low dose) Zn-DTPA/0.25 mL/100 g body weight (b.w.) 30 min before i.p. contamination with 0.037 MBq ^65^ZnCl_2_/rat.

Experiment 2 involved three groups of seven rats. Group 1 (control 2) received i.p. 0.037 MBq ^65^ZnCl_2_/rat. Group 2 received i.p. solutions containing 5 mg (high dose) Zn-DTPA/0.25 mL/100 g b.w. 30 min before i.p. contamination with 0.037 MBq ^65^ZnCl_2_/rat. Group 3 received i.p. 5 mg Zn-DTPA solutions 30 min before and 24 h after i.p. contamination with 0.037 MBq ^65^ZnCl_2_/rat.

Experiment 3 involved two groups of seven rats. Animals were contaminated i.p. on day zero with 0.037 MBq ^65^ZnCl_2_/rat. After 12 days, solutions containing 5 mg of Zn-DTPA/0.25 mL/100 g b.w. were administered i.p. for five consecutive days. The ^65^Zn retention was measured daily from day 13 to day 23.

### 2.4. Data Analysis

The retentions for each sampling time were normalized to the initial sampling time (i.e., t = 0) and calculated as percentage values. All data are presented as means ± standard errors (SEM). The relationships between the retention data and time were evaluated by linear or non-linear regression, and Pearson correlation analysis using GraphPad Prism 9.0 (GraphPad Software, Inc., San Diego, CA, USA). Comparisons among multiple groups were performed by one-way ANOVA with Newman–Keuls post hoc test, performed using GraphPad Prism 9.0. Two-tailed *p* values < 0.05 were considered statistically significant. All the pharmacokinetics parameters were evaluated using Kinetica 4.2 (InnaPhase, Inc., Philadelphia, PA, USA) software.

## 3. Results and Discussion

### 3.1. Dose Dependence of ^65^Zn Decorporation

**Figure 2:**
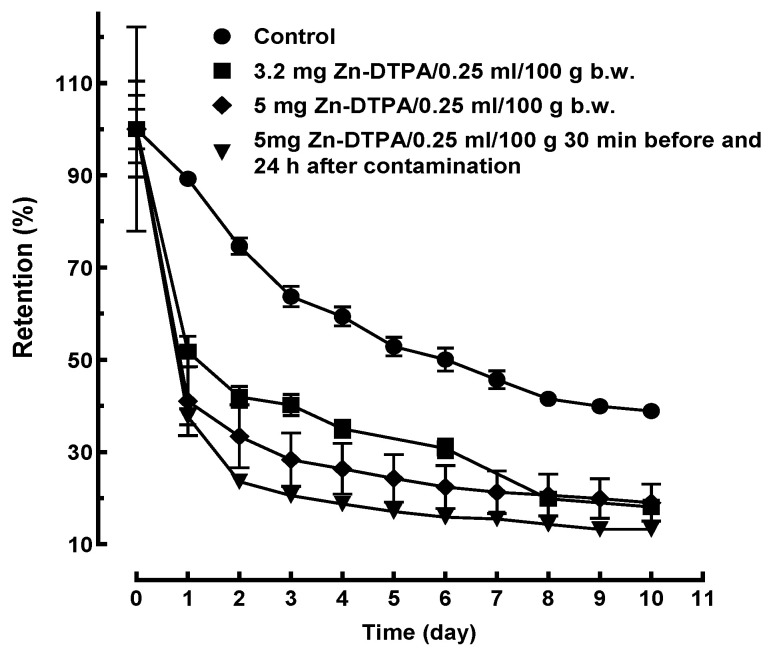
Time-course of ^65^Zn decorporation following rapid intervention with Zn-DTPA in rats. Data points represent means ± SEM (*n* = 7).

The retention data for the control groups were statistically similar for experiments 1 and 2 (data not shown). The retention data in the control group for experiment 2 being complete (i.e., for ten consecutive days), they were considered as a reference for both experiments. The decorporation of ^65^Zn by Zn-DTPA showed a remarkable time- and dose-dependence effectiveness (Figure 2).

**Figure 3:**
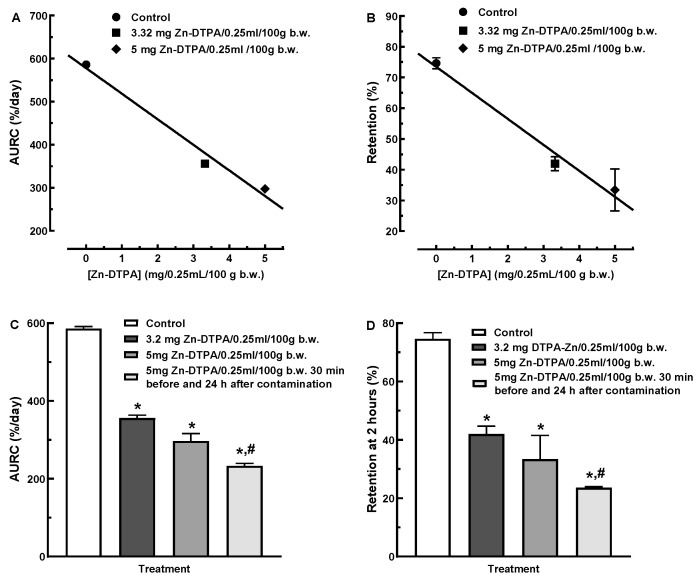
Dose-dependence of ^65^Zn decorporation on Zn-DTPA. (**A**) Linear-dependence of ^65^Zn area under the retention curve (AURC) on Zn-DTPA dose. (**B**) Linear-dependence of ^65^Zn retention on Zn-DTPA dose at two days post contamination. (**C**) Comparison of ^65^Zn AURC on Zn-DTPA treatments. (**D**) Comparison of the ^65^Zn retention on Zn-DTPA treatments at two days post contamination. Data represent means ± SEM (*n* = 7). Statistically significant difference compared to control (*) and low-dose Zn-DTPA (#), *p* < 0.05, one-way ANOVA.

A global metric used to compare the pharmacokinetics curves is the area under the plasma concentration curve [18]. Similarly to this parameter, we calculated the area under the retention curve (AURC). We found a strong linear dependence of AURCs (Figure 3A, AURC = −59.51 × [Zn-DTPA] + 578.1, R^2^ = 0.980) and %Retention (Figure 3B, %Retention = −8.46 × [Zn-DTPA] + 73.46, R^2^ = 0.981) on the Zn-DTPA dose. The retention differences after two days showed similar sensitivity to the AURC-based metric, i.e., a two-fold decrease in the contamination at the 5 mg Zn-DTPA dose (Figure 3C,D). Notably, the administration of a second higher Zn-DTPA dose after 24 h following the contamination with ^65^Zn resulted in a significant decorporation efficacy compared to a single lower dose Zn-DTPA intervention (Figure 3C,D).

### 3.2. Mono-Exponential Kinetics Modeling of ^65^ZnCl_2_ Decorporation Following Rapid Intervention with Zn-DTPA

The retention of ^65^Zn is a resultant of the DTPA and its multiple complex pharmacokinetics. It has been reported that the retention of DTPA in the blood after intravenous administration can be described by three exponential components with half-times of 1.4 min (approximately 60%), 14.3 min (approximately 20%), and 95 min (approximately 20%), respectively [19]. Our experiments evaluated the total retention of ^65^Zn in the body following Zn-DTPA treatments and the first measurement was performed at 24 h. This would make it difficult to compare our data with reported results.

**Figure 4:**
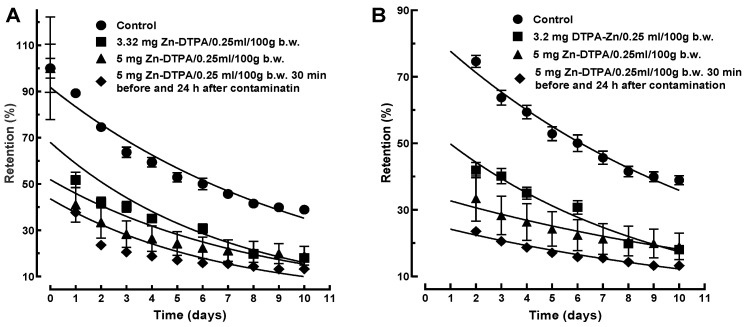
Mono-phasic exponential kinetics model of ^65^ZnCl_2_ decorporation following rapid intervention with Zn-DTPA in rats. (**A**) Mono-phasic kinetics model applied to the 0 to 10 days interval. (**B**) Mono-phasic kinetics model applied to the 2 to 10 days interval. Data represent means ± SEM (*n* = 7).

The ^65^Zn retention data following the interventions with both low and high Zn-DTPA doses were first fitted with a single exponential. The fitting was not satisfactory for the entire measurement interval (Figure 4A). However, for the Zn-DTPA treatments, the theoretical curves started from values substantially lower than 100%, which is an unsatisfactory global fitting (Figure 4A). Since this failure involves mainly the extrapolated to zero time points, we performed a single exponential fitting for the 2 to 10 days interval. This resulted only in marginal differences of the fitting performances for both low and high Zn-DTPA doses (Figure 4B), suggesting that a monophasic kinetics model is not applicable for ^65^Zn decorporation following rapid intervention with Zn-DTPA.

### 3.3. Two Mono-Exponential Steps Kinetics Model

#### 3.3.1. ^65^Zn Decorporation Modeling Following Rapid Intervention with Zn-DTPA

**Figure 5:**
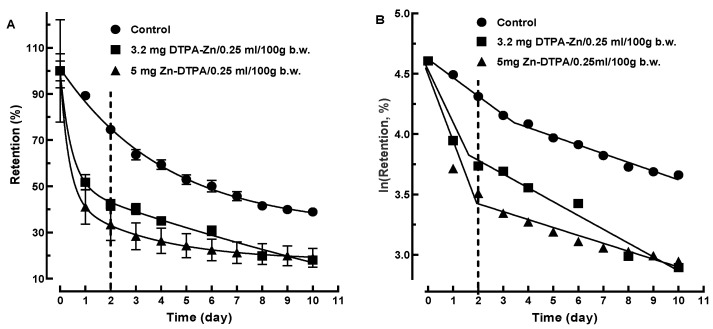
Biphasic kinetics modeling of ^65^ZnCl_2_ decorporation following rapid intervention with Zn-DTPA in rats. (**A**) Biphasic exponential time dependence of the ^65^Zn % retention. (**B**) Linear time dependence of the logarithmic transformation of ^65^Zn % retention. Data represent means ± SEM (*n* = 7).

The examination of the retention data using two-phase linear regression fitting (Figure 5B), especially in the case of the higher dose, suggests that a biphasic kinetics model [20,21] is more reliable than a single-phase modeling (Figure 5A). This is supported by the better fitting performances obtained for both the lower and higher dose of Zn-DTPA (R^2^ = 0.997 and R^2^ = 0.999, respectively). The biphasic, two mono-exponential steps kinetics model further evidenced an inflection point that appears to occur between the second and third day post contamination with ^65^Zn (Figure 5B).

#### 3.3.2. ^65^Zn Decorporation Modeling Following Delayed Intervention with Zn-DTPA

**Figure 6:**
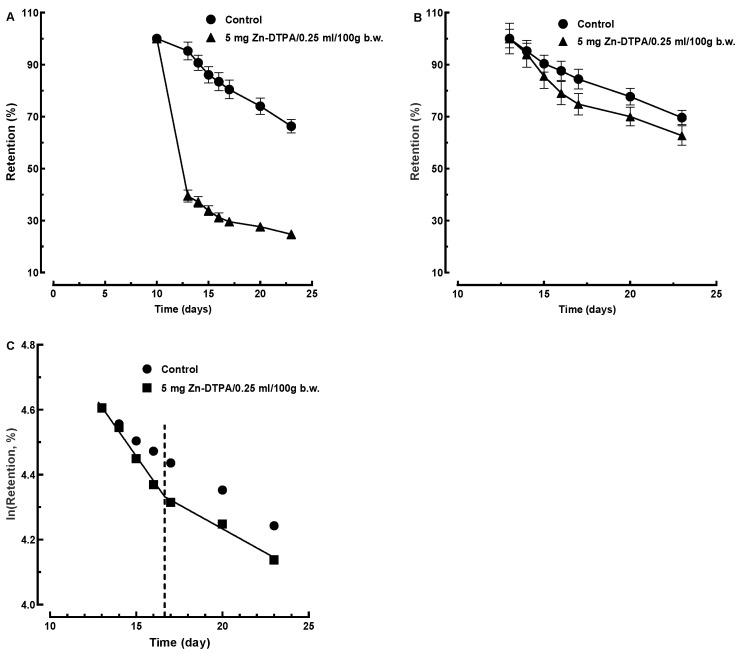
Time course and biphasic kinetics modeling of ^65^Zn decorporation following delayed Zn-DTPA intervention (i.e., after 12 days following contamination with ^65^ZnCl_2_). (**A**) Time-course of ^65^Zn decorporation following delayed Zn-DTPA intervention normalized to the retention data at day 12. (**B**) Time-course of ^65^Zn decorporation by Zn-DTPA normalized to the retention data at day 13. (**C**) Two-steps linear regression of the logarithmic transformed retention data for the 13 to 23 days interval. Data represent means ± SEM (*n* = 7).

We are reporting the efficacy of a delayed decontamination intervention by several days, following a radiotoxic contamination. On the first day of the delayed intervention with Zn-DTPA (i.e., at day 12 following contamination with ^65^ZnCl_2_), the contamination decreased to less than half (Figure 6). Repeated administration of Zn-DTPA further increased the ^65^Zn decorporation efficacy by Zn-DTPA.

The logarithmic transformation of ^65^Zn retention data suggests an exponential process during the treatment and a change to another exponential process approximately between days 17 and 23 (Figure 6C). The fitting performance was good for both time intervals (R^2^ = 0.989 and R^2^ = 0.980, respectively). Our results suggest that the elimination of ^65^Zn occurs in two phases: a rapid elimination, seemingly from blood, during the first day, followed by a slower elimination, likely concerning the radionuclide that entered the tissues. The elimination from tissues includes a transfer across cellular membranes. We could hypothesize that ^65^Zn-DTPA and other complexes of ^65^Zn formed in the blood and/or in tissues transfer across the lipid membranes from the cellular cytoplasm to the blood [22]. Further research is warranted to address the transport of Zn complexes across biological membranes.

### 3.4. Compartmental Mathematical Modeling

A model for describing the kinetics of plutonium decorporation has been reported as a mono-exponential fitting of data [23]. The ^65^Zn urinary excretion has been also found to decrease mono-exponentially as a function of time after a single dose [24]. The International Commission on Radiological Protection (ICRP) published mathematical models with a great number of compartments to describe the deposition, clearance, and dosimetry of inhaled radioactive materials in the respiratory tract [25,26]. In order to be applied, this model requires a substantial amount of data concerning the contamination of almost all organs. Although this is a commendable approach, the identification of the inter-compartmental transfer constants represents a tremendous mathematical challenge and a substantial amount of uncertainty regarding the results.

#### 3.4.1. The Bi-Compartmental Model

**Figure 7:**
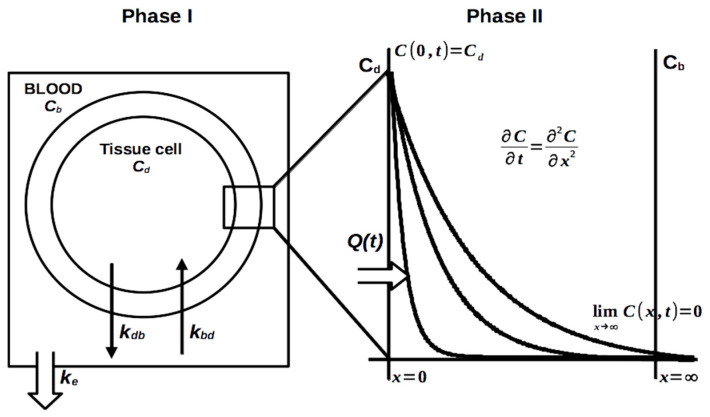
Bi-compartmental (i.e., blood to tissues) and diffusional models for ^65^Zn decorporation following Zn-DTPA intervention.

Assuming that almost the entire amount of ^65^Zn in the living body is included in a DTPA complex, we imagined the pharmacokinetics of ^65^Zn as the pharmacokinetics of the ^65^Zn-DTPA complex using a bi-compartmental model. A bi-compartmental model has been previously proposed for ^65^Zn sulfate and ^65^Zn pantothenate salts [24]. The biphasic pharmacokinetics of ^65^Zn we found in our experiments suggest the possibility of a bi-compartmental model, where the Zn complexes distribute between a “deep” compartment and the blood compartment, the elimination occurring mainly urinary (Figure 7). During the first stage, almost all ^65^Zn found is in blood and we can neglect the tissue concentration. At a longer time after administration, the amount in blood is very small and is practically due to the slow transfer from the “deep” tissue pool.

The associated equations of the model are Equations (1) and (2) (Figure 7, phase I).

(1) $$\frac{dC_{b}}{dt}=-\left(k_{bd}+k_{e}\right)C_{b}+k_{db}C_{d}$$

(2) $$\frac{dC_{d}}{dt}=k_{bd}C_{b}-k_{db}C_{d}$$

With the initial conditions *C_b_*(0) = *C*_0_ and *C_b_*(0) = 0, the mathematical solution for the blood concentration (*C_b_*) is

(3) $$C_{b}\left(t\right)=\frac{C_{0}\left(k_{db}-\beta \right)}{\alpha -\beta }e^{-\beta t}+\frac{C_{0}\left(k_{db}-\alpha \right)}{\beta -\alpha }e^{-\alpha t}$$

where *α* and *β* are the eigenvalues of the matrix of coefficients obtained after the application of the Laplace transform. The equations were written in concentrations. However, after multiplying with the volume of distribution of both members, the equations become a relationship between the amounts, the coefficients remaining unchanged.

**Figure 8:**
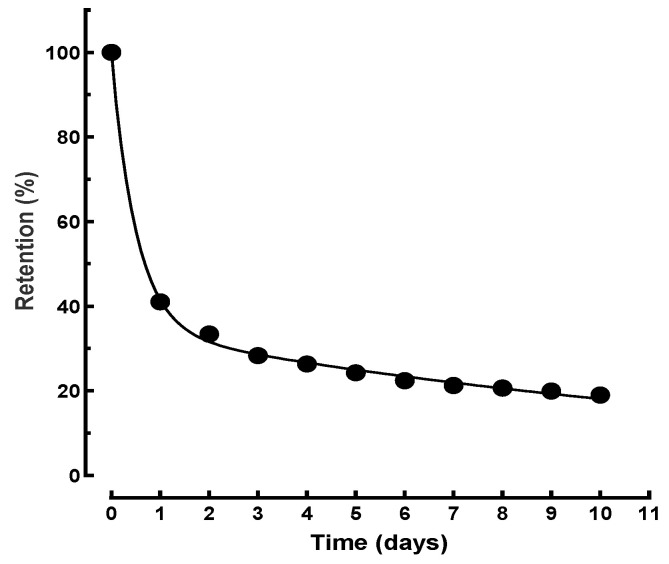
Pharmacokinetics mathematical modeling of the ^65^Zn decorporation following rapid intervention with higher dose of Zn-DTPA (i.e., 5 mg/0.25 mL/100 g b.w.).

The fitting of the experimental data with the solution of the bi-compartmental model, particularly for the contamination with ^65^Zn before the administration of 5 mg Zn-DTPA dose, was excellent (Figure 8). Calculating the transfer constants from *A*, *B*, *α*, and *β*, we obtained the following rate constants for the transfers of the ^65^Zn complex between compartments: *k_bd_* = 1.77 day^−1^, *k_db_* = 1.21 day^−1^. Similarly, we calculated the following elimination constant for the ^65^Zn complex: *k_e_* = 0.19 day^−1^, and the corresponding elimination half-time: *t*_1/2_ = (ln2)/0.19 = 3.6 days.

A comparison between total ^65^Zn pharmacokinetics in control and treated animals is primarily a comparison among ^65^Zn, the Zn-DTPA complex, and zinc-albumin and zinc-amino acid complexes. The zinc-amino acid complexes can be passively transported through tissue membranes to bind to proteins [27]. However, the differences are especially apparent in the early days. As evidenced in Figure 5, the elimination rates in the following days are very similar. The calculations in the case of control data gave *t*_1/2_ = 2.8 days and for the last points *t*_1/2_ = 81 days. In the first part of the experiment, the model suggests a much slower transfer between compartments than in the present Zn-DTPA: *k_bd_* = 0.14 day^−1^, *k_db_* = 0.09 day^−1^.

#### 3.4.2. Degeneration of the Bi-Compartmental Model

Our two-compartment model describes well the evolution of the ^65^Zn retention for the entire time interval. Since after a short period of time (i.e., first phase) the ^65^Zn complexes from blood are eliminated and a significant amount of ^65^Zn remains in the “deep” tissue. This amount would represent a source of ^65^Zn complexes for the blood. Consequently, the model becomes approximately *C_d_(t) ≈ C*_*d*0_ *e^−k^_db_^t^* (where, *C*_*d*0_ is the starting concentration/activity in tissue cells), which is a mono-exponential process. This exponential is determined, however, by the transfer from the “deep” compartment to the blood and not by the renal elimination. In the second phase (i.e., after removing a large part of ^65^Zn on the first day), the “deep” compartment acquires the role of the “central compartment”. The elimination in the first phase refers to the ^65^Zn complex in the blood and, in the second phase, the elimination refers to the ^65^Zn complex initially distributed in the “deep” compartment, which is slowly transferred back into the blood.

### 3.5. The Diffusion Model. The Square-Root Law

We further investigated an alternative kinetics model to describe the time-course of the ^65^Zn retention for the 2 to 10 days interval. The model proposed previously [28,29] is based on the hypothesis that the elimination process from cells is controlled by the diffusion inside the lipid cell membrane (Figure 6), a process described by the Fick’s diffusion equation [Equation (4)].

(4) $$\frac{\partial C}{\partial t}=D\frac{\partial^{2}C}{\partial x^{2}}$$

As phenomenological conditions, we considered that the tissue cells behave as an “infinite” reservoir with a constant concentration of *C_d_*_0_, similar to a thermostat in the theory of heat transfer [30]. The transfer occurs across membranes. Since a Zn complex has higher solubility in water, its escape from membrane to plasma is rapid. The rate determining process is the diffusion inside the membranes. The associated mathematical problem is to solve the diffusion equation with the initial and boundary conditions *C*(0,*t*) = *C*_*d*0_, *C*(*x*,0) = 0, $\underset{x\to \infty }{\lim}C\left(x,t\right)=0$ (Figure 7, phase II).

The mathematical solution can be obtained in the form of Equation (5).

(5) $$C\left(x,t\right)=C_{d0}\left[1-\int_{0}^{\frac{x}{\sqrt{4Dt}}}e^{\frac{-u^{2}}{2}}du\right]$$

Calculating the Zn complex flux *J* across the membrane’s inner interface (i.e., *x* = 0) and integrating it as a function of time, we obtain the mathematical Equation (6) for the quantity *Q*(*t*) of the Zn complex that is transferred across cell membranes after a certain period of the time *t*.

(6) $$Q\left(t\right)=A\int_{0}^{t}Jdt=\frac{2AC_{d0}}{\sqrt{\pi }}\sqrt{Dt}$$

where $J=-D\frac{\partial C}{\partial x}\left(0,t\right)$; and *D*, *x*, and *A* are, respectively, the diffusion coefficient, the distance from the interface and the interface area. (We actually measured %Retention = 100 ∗ (initial activity in the deep compartment − *Q*(*t*))/initial activity), which satisfies the same equation.)

#### 3.5.1. Transfer Modeling Following Rapid Intervention (Days 2 to 10)

**Figure 9:**
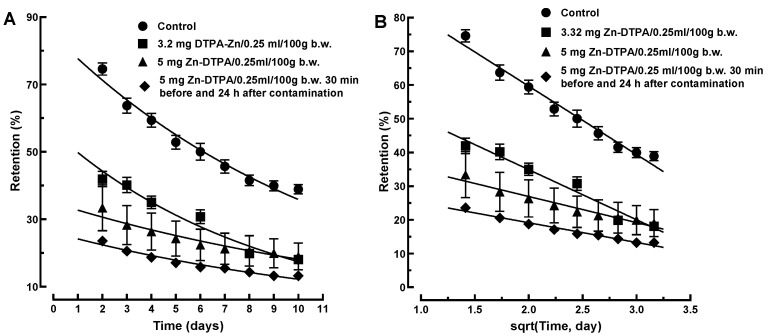
Comparison of the diffusional kinetics model and mono-phasic kinetics model for ^65^Zn decorporation following rapid intervention with Zn-DTPA. (**A**) Mono-phasic exponential kinetics model of ^65^Zn decorporation following rapid intervention with Zn-DTPA. (**B**) Square-root diffusional model of ^65^Zn decorporation following rapid intervention with Zn-DTPA. Data represent means ± SEM (*n* = 7).

In this model, the amount of the complex *Q*(*t*) transferred across the interface A is proportional to the square-root of time. If the elimination process is diffusion controlled, a good linear dependence of the experimental on the square-root of time is expected to be obtained. Indeed, the square-root diffusional model described better our experimental data when compared to that of the mono-exponential model (Figure 9A,B). The correlation coefficients obtained for the diffusional kinetics model, when compared to the mono-exponential kinetics model, were consistently better for either the low- (R^2^ = 0.964 versus R^2^ = 0.961) or high-dose of Zn-DTPA (R^2^ = 0.951 versus R^2^ = 0.932), as well as for the 24 h repeated high-dose of Zn-DTPA (R^2^ = 0.970 versus R^2^ = 0.961).

#### 3.5.2. Long-Term ^65^Zn Decorporation Modeling Following Delayed Intervention with Zn-DTPA

**Figure 10:**
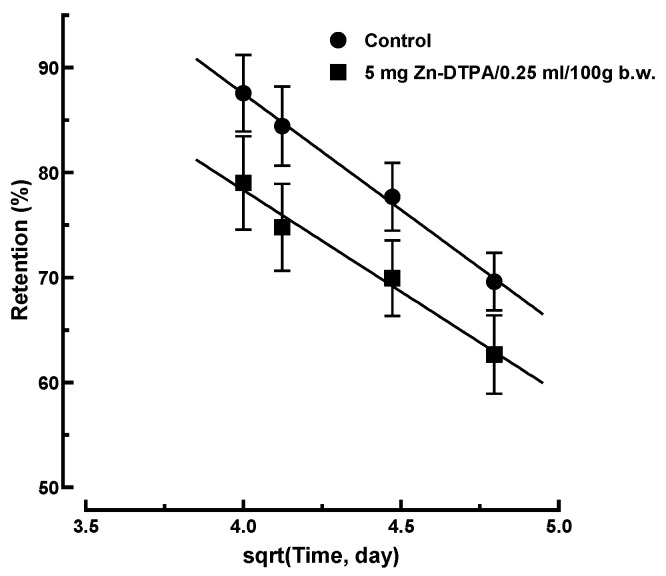
Square-root law modeling of ^65^Zn retention following delayed intervention with Zn-DTPA for the 16 to 23 days interval compared to the control group. Data represent means ± SEM (*n* = 7).

It is noteworthy that the controlled release from tissues by diffusion appears to be an increasingly reliable model as the process progresses over time. The predictions of the square-root model for the control group and the group treated with the higher dose of Zn-DTPA showed a remarkable fitting performance for the delayed intervention (Figure 10, %Retention = -19.4 × sqrt[Time, day] + 156, R^2^ = 0.983) when compared with the untreated group (Figure 10, %Retention = −22.1 × sqrt[Time, day] + 176, R^2^ = 0.997).

Zn(DTPA) distributes from blood mainly in the extracellular fluid, and enters cells slowly and to a limited extent, explaining how radionuclide-DTPA complexes may be excreted over several weeks. The justification for considering a mono-compartmental kinetics model for the decorporation of ^65^Zn arises from the fact that radionuclides pass through membranes with difficulty and their concentration in tissues is very low compared to its concentration in the blood, at least in the first day after contamination. The elimination from the blood being preponderant compared to the elimination from tissues, a mono-exponential decrease in the radionuclide in the living body is an expected result. Since the elimination from blood is rapid, the elimination beyond 24 h concerns mainly the amount remained in the tissues, which is slowly transferred to blood.

Our results suggest that the elimination in the first day is a mono-exponential process and may explain the rapid decrease in the radiotoxic nuclide in the body (Figure 2). However, we did not clearly evidence this due to the particular scheme of our sampling method (i.e., one per day). In fact, the estimated elimination constants are strongly dependent on the time intervals to which the modeling refers (e.g., hours, days, or weeks), what was measured, and where the measurements were performed. The interpretations aim to be reliable, the mnemonic hypotheses to encompass the multitude of data.

In a second phase, the ^65^Zn complexes diffuse from cells through the membrane into the blood and are further eliminated, likely by glomerular filtration. The kinetics of the whole process is given by the rate determining step, which is the diffusion through the cell membranes. The second compartment conceivably includes tissues, even though the uptake mechanism is not clear. Nevertheless, our results can be explained with a bi-compartmental model for the first part of the experiment and its degeneration to a mono-compartmental model in the second part. A criticism of the above models could be that they are only partial physiological models.

Very complex physiological models concerning the distribution evolution of radionuclides in the environment and living body (e.g., “biokinetic” models) have been recommended by the ICRP [31]. The task group “Research studies on biokinetic models” proposed the simultaneous kinetics evaluation of the radionuclide, as well as of the decorporator and their complexes [32]. These models have the disadvantage of being based on mean and simulated data and the identification of their parameters encounters a number insurmountable mathematical difficulties. For example, subsequent research failed to confirm the possibility of data extrapolation for Pu-DTPA complexes to long-term decorporation efficacy [33], which appears to argue for an early intervention with the chelation therapy following radiotoxic contamination [34].

## 4. Conclusions

In all cases (i.e., control and Zn-DTPA solutions), the kinetics of the ^65^Zn decorporation included two different evolutions of the whole-body radioactivity. In the first day, there appeared to be an abrupt decrease in the radioactivity followed by a slow, exponential decrease during the next days. Considering that in the living body the ^65^Zn complexes undergo numerous transfers across interfaces and diffusions, the modeling of the data can be performed based on a first-order kinetics and on a diffusion square-root law model. The relatively rapid change from a bi-exponential to a mono-exponential process is explained as a degeneration of the model due to a higher rate of renal elimination of the ^65^Zn-DTPA complex, rather than the transfers between a “central” compartment (e.g., the blood) and a “deep” compartment (e.g., tissues).

Splitting data and modeling into two steps using different models or the same model, but with different parameters, allows a better understanding, description, and prediction of the evolution of contamination of the whole body.

The square-root law model fitted better our experimental data compared to the mono-compartmental model in the second phase of experiment. This allows us to argue for a diffusion-controlled transfer across membranes from tissues into the blood, and for a general square-root law to describe the ^65^Zn decorporation kinetics following Zn-DTPA administration as a diffusion-controlled process from an “infinite” reservoir into a “semi-finite” medium. This law also predicts a slower release of the radionuclide from tissues compared to the exponential or multi-exponential equations and could better explain the very long persistence of radionuclides in the living body.

Our experiments and mathematical modeling highlight, as a general aspect regarding the decorporation of radionuclides from the living body, the necessity to split the interventions into two types of treatments: (i) a treatment for the elimination of radionuclides from the blood, a very short-term treatment, by an immediate intervention; and (ii) a treatment for the removal of radionuclides from tissues, a long-term treatment, by a late intervention, even without an end.

## Data Availability

The data presented in this research are available upon request from the corresponding author C.M.
